# Structural Characterization and Anti-Colitis Mechanisms of *Polygonatum sibiricum* Polysaccharides via Modulation of Neutrophil Extracellular Traps (NETs)—Macrophage Crosstalk

**DOI:** 10.3390/nu18071046

**Published:** 2026-03-25

**Authors:** Jiaman Xu, Junna Zheng, Wukang Ke, Yu Qiu, Lu Zhang, Chenxi Wu, Xiaoxi Zhang, Daozong Xia, Fenfen Li

**Affiliations:** 1 School of Pharmaceutical Sciences, Zhejiang Chinese Medical University, Hangzhou 310053, China; 2 School of Life Science, Zhejiang Chinese Medical University, Hangzhou 310053, China; 3 Academy of Chinese Medical Sciences, Zhejiang Chinese Medical University, Hangzhou 310053, China

**Keywords:** *Polygonatum sibiricum*, polysaccharide, structural characterization, macrophage, neutrophil extracellular traps, inflammation

## Abstract

Background: *Polygonatum sibiricum* (PS), a perennial herbaceous plant belonging to the Liliaceae family, is widely distributed in China and other East Asian countries. PS has been used as food and medicine for thousands of years, and its rhizomes are rich in *Polygonatum sibiricum* polysaccharides (PSP), which exhibit various bioactivities, yet their structural features and therapeutic mechanisms against ulcerative colitis (UC) remain unclear. Methods: A homogeneous polysaccharide, PSP-1b (57.45 kDa), was isolated from the rhizomes of PS via ion-exchange and gel filtration chromatography and structurally characterized using chromatographic and spectroscopic methods. In vivo, its effects were evaluated in a dextran sulfate sodium (DSS)-induced mouse model of UC, while in vitro mechanisms were explored using macrophages stimulated with lipopolysaccharide (LPS) and neutrophil extracellular traps (NETs). Results: PSP-1b was identified as a neutral polysaccharide with minimal branching. Its primary structural backbone was largely composed of →4)-β-D-Gal*p*-(1→ residues. A portion of these backbone residues was substituted at the O-6 position by side chains primarily composed of β-D-Gal*p*-(1→ units. In vivo, PSP-1b significantly alleviated DSS-induced colitis by reducing inflammatory cytokine secretion, suppressing colonic macrophage infiltration, and reversing neutrophil extracellular traps (NETs) deposition. In vitro, PSP-1b directly interacted with TLR4, inhibited the MAPK/NF-κB signaling pathway, and attenuated LPS- and NET-induced macrophage polarization and inflammation. Conclusions: PSP-1b as a promising candidate for functional foods or therapeutic agents targeting inflammatory bowel disease.

## 1. Introduction

Ulcerative colitis (UC) is a refractory inflammatory bowel disease characterized by persistent colonic mucosal inflammation, with primary clinical manifestations including diarrhea, hematochezia, and abdominal pain [1]. While the exact etiology of UC remains incompletely understood, it is thought to involve genetic susceptibility, immune dysregulation, and intestinal dysbiosis [2]. Primary clinical interventions include pharmacotherapy (e.g., glucocorticoids and aminosalicylate) [3], surgical management (e.g., partial colectomy) [4], and cellular therapies (e.g., stem cell transplantation and leukocyte adsorption therapy) [5]. Despite their established efficacy, these approaches are frequently associated with considerable adverse effects, a high likelihood of relapse after treatment cessation, and inherent procedural risks. Consequently, increasing emphasis has been placed on traditional Chinese medicine (TCM) for UC management, with studies indicating favorable therapeutic outcomes and minimal adverse reactions associated with TCM treatment [6]. TCM is grounded in the rich theoretical framework and practices of Chinese culture, with a history of effective treatments that spans over 2000 years. Numerous classical herbal formulations have been widely applied in TCM clinical practice to alleviate gastrointestinal dysfunction and inflammation [7]. According to historical Chinese medical texts, Shenling Baizhu Powder, Baitouweng Decoction, Shaoyao Decoction, and Lizhong Decoction are classic traditional Chinese medicine formulas with a long history of clinical application in treating UC. Shenling Baizhu Powder, first recorded in the Taiping Huimin Heji Jufang of the Song Dynasty, strengthens the spleen and stops diarrhea [8]. Baitouweng Decoction, originating from Zhang Zhongjing’s Treatise on Cold Damage Disorders, clears heat, resolves toxicity, and relieves dysentery [9]. Shaoyao Decoction, documented in Suwen Bingji Qibao Mingji (Jin Dynasty), clears damp-heat, regulates qi, and promotes blood circulation [6]. Lizhong Decoction, also described in Shanghan Lun, exerts anti-oxidative [10] and anti-inflammatory [11] effects. Modern pharmacological studies have demonstrated that these formulas alleviate UC symptoms in mice, ameliorate DSS-induced colon shortening and intestinal mucosal damage, and reduce inflammatory cytokine levels, collectively supporting their therapeutic efficacy in UC. Their therapeutic advantages are attributed to TCM’s characteristic multi-component, multi-target, and holistic regulatory mechanisms, which collectively aim to deliver efficacy while minimizing adverse effects. Consequently, TCM has become a prominent focus in contemporary UC research.

*Polygonatum sibiricum* (PS) is the dried rhizome of *Polygonatum kingianum* Coll. Et Hemsl., *Polygonatum sibiricum* Red., or *Polygonatum cytomema* Hua., which has a sweet fragrance and taste, and is well-known as a traditional medicinal herb and functional food in China [12]. Polysaccharides are the main bioactive substances found in the rhizome of PS [13]. In recent years, pharmacological studies have shown that *Polygonatum sibiricum* Polysaccharides (PSPs) exhibit various beneficial effects, including antioxidant properties [14], anti-inflammatory effects [15], immune enhancement [16], and anti-tumor effects [17]. Additionally, as an immunostimulatory polysaccharide, PSP activates the host immune system through interactions with immune cells. Multiple studies have demonstrated that PSP significantly reduces mortality in septic mice. It also decreases myeloperoxidase (MPO) activity, a marker of neutrophil infiltration in the liver [18]. Furthermore, PSP restores neutrophil and macrophage populations to normal levels and significantly suppresses the secretion of pro-inflammatory factors [19].

During UC pathogenesis, neutrophil extracellular traps (NETs) contribute to inflammatory cascades [20]. NETs are net-like structures released by neutrophils in response to recognition and stimulation by invading pathogens. They contain various granular proteins such as DNA, citrullinated histones (CitH3), neutrophil elastase (NE), and MPO. Studies have shown that neutrophil activation and NET deposition occur in the colonic mucosa of both UC patients and animal models [21]. NETs contribute to the disruption of the intestinal epithelial barrier by impairing tight junctions and inducing apoptosis, thereby increasing intestinal permeability [22]. Concurrently, Gram-negative bacteria, which possess lipopolysaccharide (LPS) in their outer membranes, show increased abundance in UC. Impaired barrier function facilitates LPS translocation, which activates immune cells and exacerbates intestinal inflammation [23,24]. Furthermore, NET components activate macrophages via TLR signaling, promoting the activation of NF-κB/MAPK pathways and stimulating the release of large quantities of inflammatory cytokines. These cytokines form a positive feedback loop with TNF-α and IL-1β, further amplifying the inflammatory response [25,26]. However, therapeutic strategies for UC from the perspective of targeting NET–macrophage interactions have not yet been clarified.

While our prior work established a link between PSP’s efficacy and NET modulation in colitis, the precise molecular mechanisms underlying this modulation, particularly the intricate crosstalk between NETs and macrophages, and the attribution of these effects to specific, defined PSP components remain largely unexplored. To address this, we isolated and purified polysaccharide fractions from PS, characterized their structural properties, and evaluated their anti-UC efficacy in vivo and in vitro. Furthermore, we explored the therapeutic mechanisms of PS-derived polysaccharides in alleviating colitis through the lens of NET–macrophage crosstalk. This work aims to provide novel therapeutic strategies for UC and establish a scientific foundation for further research on PSP.

## 2. Materials and Methods

### 2.1. Materials and Reagents

Steamed PS was obtained from Quzhou, China. DEAE Sepharose FF and Sephadex G100 were purchased from Cytiva (Marlborough, MA, USA). Dialysis bags (MWCO: 3500 Da and 8000–14,000 Da) were sourced from Shanghai Yuanye (Shanghai, China). LPS was acquired from Sigma-Aldrich (St. Louis, MO, USA), which also supplied D-galactose, D-glucose, and other monosaccharide standards. A series of dextrans with narrow molecular weight distribution was obtained from the National Institute of Metrology, Beijing, China, and Dextran sulfate sodium (DSS) was from MP (Las Vegas, NV, USA).

Antibodies for p-ERK, ERK, p-p38, p38, and p65 were supplied by CST (Danvers, MA, USA). Diagbio (Hangzhou, China) provided antibodies for p-JNK, JNK, and β-tubulin. TLR4 Mouse mAb was purchased from Bioss (Beijing, China), and GAPDH from Proteintech (Wuhan, China). MPO and CitH3 were purchased from ABCAM (Cambridge, UK). F4/80 Rabbit mAb and neutrophil elastase (ELANE) Rabbit pAb were obtained from ABclonal (Wuhan, China). TSA kits were purchased from CELNOVTE (Rockville, MD, USA). Dimethylsulfoxide (DMSO) was obtained from Solarbio (Beijing, China). iFluo^TM^ 488-conjugated goat anti-rabbit IgG polyclonal antibody was provided by Huabio (Hangzhou, China). FITC anti-mouse F4/80 antibody and PE anti-mouse CD86 antibody were purchased from Biolegend (San Diego, CA, USA).

ELISA kits for IL-6, TNF-α, IL-10, IL-12(p70), and IL-13 were obtained from Boster (Wuhan, China). BCA and lactate dehydrogenase (LDH) kits were purchased from Beyotime (Shanghai, China).

### 2.2. Extraction and Purification of PSP

PSPs were extracted using hot water followed by alcohol precipitation. Briefly, PS was first dried and ground into a fine powder. Subsequently, it was immersed in 95% ethanol overnight to remove lipids and pigments, followed by two rounds of extraction with hot deionized water (material-to-liquid ratio of 1:20, *w*/*v*, 95 °C). The combined extracts were then precipitated by adding 95% ethanol to a final concentration of 80% (*v*/*v*) and allowed to stand at 4 °C overnight. After centrifugation at 3500 rpm for 15 min, the precipitate was redissolved in water. Proteins were removed using the Sevag method [27], and pigments were eliminated using activated carbon. Then, the polysaccharides were dialyzed using a 3500 Da molecular weight cutoff membrane and freeze-dried to obtain PSP. An appropriate amount of polysaccharide powder was dissolved and loaded onto a DEAE FF column (1.6 × 60 cm). Elution was performed using a 0.0–0.5 M NaCl gradient at 2 mL/min, and fractions were collected at 6 mL/tube. The carbohydrate content of each tube was measured. The solutions were collected based on the elution curve and were designated as PSP-1, PSP-2, PSP-3, and PSP-4. Subsequently, a Sephadex G100 column (1.6 × 80 cm) was used to further separate the polysaccharides PSP-1, PSP-2, and PSP-3. Elution was conducted with ultra-pure water, resulting in the isolation of four purified polysaccharides designated as PSP-1a, PSP-1b, PSP-2a, and PSP-3a.

### 2.3. Structural Analysis of PSP-1b

#### 2.3.1. Chemical Composition Analysis

Total carbohydrate content was determined using an improved phenol–sulfuric acid method with Glc as the standard [28]. Protein content was measured using the Coomassie Brilliant Blue G-250 technique at a wavelength of 590 nm [29].

#### 2.3.2. Molecular Weight Measurement

According to a previously published method, HPGPC was carried out to analyze the molecular mass and homogeneity of PSP-1b. HPGPC analysis was performed using a size-exclusion chromatography (SEC) system composed of a Waters 1515 isocratic HPLC pump, a Waters 2707 autosampler, a Waters 2414 refractive index detector, and Ultrahydrogel series columns (Waters, Milford, MA, USA). An aqueous solution containing 0.1 mol/L NaNO_3_ was employed as the eluent at 1 mL/min at 35 °C. Dextran standards were used for calibration [30].

#### 2.3.3. Monosaccharide Composition Analysis

The monosaccharide composition of PSP-1b was determined via complete acid hydrolysis [31], followed by high-performance anion-exchange chromatography (HPAEC) using a Dionex ICS 5000 system configured with a CarboPac™ PA-20 column (150 × 3.0 mm) and a pulsed amperometric detector (PAD) (Thermo Fisher Scientific, Waltham, MA, USA).

#### 2.3.4. UV–Vis Absorption and FT-IR Spectroscopy Analyses

PSP-1b was analyzed for the presence of proteins and nucleic acids using a UV–vis spectrophotometer (SHIMADZU, Kyoto, Japan). The method was based on their distinct UV absorption characteristics, encompassing a spectral scan from 200 to 400 nm. The respective absorbances at 280 nm (proteins) and 260 nm (nucleic acids) were recorded for identification and quantification [32].

The sample was characterized using FT-IR. The transmission spectrum of the sample was recorded over the spectral range of 4000–400 cm^−1^ using a FT-IR spectrometer [33].

#### 2.3.5. Scanning Electron Microscope

The morphology of PSP-1b was observed using field emission scanning electron microscopy (HITACHI, Tokyo, Japan) [34]. The sample was examined at magnifications of 300×, 500×, 2000×, and 5000× after being sputter-coated with gold.

#### 2.3.6. Triple-Helical Conformation Analysis

The Congo red assay were performed according to the methods reported in the relevant literature [35], and λmax was determined by scanning the samples over a wavelength range of 400–700 nm.

#### 2.3.7. Methylation Analysis

PSP-1b was methylated following the method described in a previous study [36]. The fully methylated samples were hydrolyzed, reduced, and acetylated to generate partially methylated alditol acetates (PMAAs) [37]. Upon completion of the reaction, the PMAAs were obtained and finally analyzed via GC–MS.

#### 2.3.8. NMR Analysis

PSP-1b was dissolved in D_2_O (99.8% D) and then freeze-dried to facilitate deuterium exchange. NMR spectra were recorded with a Bruker Avance III 600 MHz Digital NMR Spectrometer (BRUKER, Billerica, MA, USA) at 25 °C. Chemical shift was reported in ppm.

### 2.4. Anti-Inflammatory Activity Analysis

#### 2.4.1. Animals and Experimental Design

All experimental procedures involving animals were conducted in accordance with the NIH Guidelines for the Care and Use of Laboratory Animals and were approved by the Animal Care and Use Committee of Zhejiang Chinese Medical University (SYXK 2021-0012). Male Balb/c mice (21–22 g, 6–8 weeks old) were purchased from Zhejiang Vital River Laboratory Animal Technology Co., Ltd. (Jiaxing, China). Upon arrival, all animals were kept under standard laboratory conditions (humidity: 55 ± 10%; temperature: 22 ± 2 °C) with free access to food and water.

After 7days of acclimatization, mice were randomly assigned to 3 groups (n = 6): Control, DSS, and DSS + PSP (800 mg/kg). For colitis induction, mice in the DSS and DSS + PSP groups received 3% DSS in drinking water continuously for one week, followed by 3 days of sterilized water. Meanwhile, mice in the DSS + PSP (800 mg/kg) group were intragastrically administered PSP solution (800 mg/kg·bw) throughout the experiment, while the other groups received equal amounts of sterilized water. Twenty-four hours post the final treatment, mice were euthanized, and colons were collected.

#### 2.4.2. Cell Culture

RAW 264.7 cells and HL-60 cells were purchased from the Cell Bank of the Chinese Academy of Sciences and maintained at the conditions of 37 °C and 5% CO_2_. Cells were cultured in high-glucose DMEM (RAW 264.7) or RPMI 1640 (HL-60) medium, both containing 10% fetal bovine serum (FBS), 100 U/mL penicillin, and 100 mg/mL streptomycin.

#### 2.4.3. Isolation of NETs

dHL-60 cells, which were differentiated from HL-60 cells with 1.3% DMSO for 5 days, were treated with 100 nM PMA for 4 h to induce NET formation [38,39]. Following this, NETs were extracted according to established literature protocols [40]. DNA concentration of the sample was determined using a Nanodrop 2000 (ThermoFisher, Waltham, MA, USA) at 260/280 nm [41].

#### 2.4.4. Cell Viability Assessment

RAW 264.7 cells (8 × 10^3^ cells/well) were seeded in a 96-well plate and treated with PSP-1b, PSP-2a, or PSP-3a at concentrations of 0–500 μg/mL for 24 h. Subsequently, 10 μL of CCK-8 reagent was added to each well, and the cells were incubated for 2 h prior to measuring absorbance at 450 nm.

#### 2.4.5. Measurement of LDH Levels in RAW 264.7 Cells

RAW 264.7 cells (8 × 10^3^ cells/well) were seeded in 96-well plates for 24 h of adherence. The cells were then treated with PSP-1b, PSP-2a, and PSP-3a (125 μg/mL each) in the presence of LPS (1 μg/mL) for 24 h to assess the anti-inflammatory efficacy of PSP fractions. Subsequently, LDH release was determined in accordance with the manufacturer’s instructions.

#### 2.4.6. ELISA

RAW 264.7 cells (5 × 10^5^ cells/well) were seeded in 6-well plates for 24 h of adherence. The cells were treated with PSP-1b, PSP-2a, and PSP-3a (125 μg/mL each) in the presence of LPS (1 μg/mL) for 24 h to assess their anti-inflammatory potential. For the mechanistic investigation of PSP-1b-mediated UC alleviation, cells were co-stimulated with PSP-1b (125 μg/mL), NETs (3 μg/mL), DNase I (100 U/mL), and LPS (10 ng/mL) for 6 h. The expression levels of TNF-α, IL-12 (p70), IL-6, IL-10, and IL-13 in the supernatant were determined using ELISA kits in accordance with the manufacturer’s instructions.

To quantify cytokine levels in colon tissue, samples were homogenized in ice-cold PBS, followed by centrifugation to collect the supernatant and determination of the total protein concentration. Subsequently, cytokine levels were determined with commercial ELISA kits following the manufacturers’ protocols and normalized to the total protein content.

#### 2.4.7. Flow Cytometry Analysis

RAW 264.7 cells were grouped and treated as described in Section 2.4.6. Following 24 h of stimulation, macrophage polarization was analyzed. The cells were then incubated with F4/80-FITC antibody and CD86-PE antibody for 30 min, followed by washing with PBS. Surface expression of CD86 was analyzed using Flow cytometry (BD Biosciences, San Jose, CA, USA) [42]. Data were analyzed using FlowJo software V10.

#### 2.4.8. Western Blotting

RAW 264.7 cells were grouped and treated as described in Section 2.4.6. Following 3 h of stimulation. total cellular proteins were extracted, quantified, and subjected to SDS-PAGE. Following transfer onto PVDF membranes and blocking, the blots were incubated with the corresponding primary antibodies and HRP-conjugated secondary antibodies, diluted according to the manufacturer’s instructions, followed by detection with a high-sensitivity ECL kit.

#### 2.4.9. Histological Characterization

The embedding and sectioning of colon samples followed established protocols from the literature [43]. After deparaffinization and rehydration according to standard protocols, the sections were stained employing hematoxylin and eosin (H&E) before being viewed under a light microscope. Histological evaluation was performed using a four-point scoring system that assessed multiple parameters, including mucosal ulceration, crypt damage, inflammatory cell infiltration, inflammatory exudate, fibrosis, and lesion extent, with detailed criteria provided in Table S2 [9].

#### 2.4.10. Immunofluorescence Staining

RAW 264.7 cells (2 × 10^5^ cells/well) were seeded on sterile coverslips in a 12-well plate pre-equilibrated with 100 μL of PBS. After 24 h of adherence, cells were grouped and treated as described in Section 2.4.6. Post-stimulation, the supernatant was discarded, followed by cell fixation, permeabilization, blocking, and incubation with the corresponding primary and secondary antibodies. Cell nuclei were then stained with DAPI [44]. Finally, cells were mounted with antifade mountant and imaged using a laser confocal microscope (Zeiss LSM880, Jena, Germany). Three representative images were acquired per group.

Paraffin-embedded colon sections were prepared for immunofluorescence staining. Following deparaffinization and rehydration, antigen retrieval was performed using a microwave-assisted method. After blocking, the sections were incubated overnight at 4 °C with primary antibodies against F4/80, MPO, NE, and CitH3. After washing, the sections were incubated with the corresponding fluorescence-conjugated secondary antibodies for 1 h at room temperature in the dark. Finally, cell nuclei were counterstained with DAPI prior to visualization using the Hamamatsu MoxiePlex multispectral imaging system, with three random fields captured per sample. Image analysis and quantification were carried out using ImageJ software (v1.54p).

#### 2.4.11. Cellular Thermal Shift Assay (CETSA)

The experimental method was adapted from previous literature with slight modifications [45,46]. Briefly, soluble protein lysates from RAW 264.7 cells were dispensed into PCR tubes and pretreated with PSP-1b (500 μg/mL) or sterile water at 37 °C for 30 min. The solution was then subjected to thermal denaturation at gradient temperatures (50–75 °C) for 3 min and then cooled on ice at 4 °C for an additional 3 min. After centrifugation at 12,500 rpm for 15 min at 4 °C, the soluble supernatant was subjected to Western blotting.

#### 2.4.12. Statistical Analysis

All data are presented as mean ± standard error of the mean (SEM). Statistical analyses were performed using GraphPad Prism 10.3 software. Comparisons among multiple groups were conducted using one-way analysis of variance (one-way ANOVA) followed by Tukey’s post hoc test for data with a single independent variable. For data involving two independent variables, two-way ANOVA was employed, followed by Bonferroni’s post hoc test. Notably, *p* < 0.05 was considered statistically significant.

## 3. Results

### 3.1. Preparation and Purification of PSP-1b

**Figure 1:**
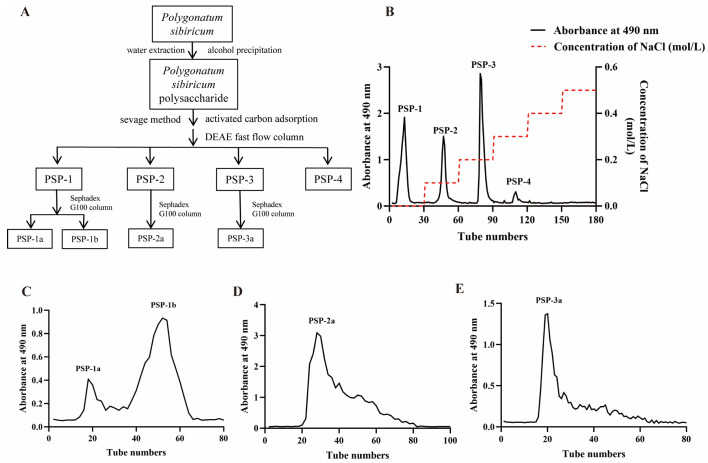
Preparation and purification of PSP. (**A**) Schematic representation of the extraction procedure. (**B**) Elution curve of crude polysaccharide using water and 0.1–0.5 M NaCl. (**C**–**E**) Elution profile of PSP-1 (**C**), PSP-2 (**D**), and PSP-3 (**E**) fractions from a Sephadex G100 column (1.6 × 80 cm) eluted with H_2_O.

The whole process of extracting and purifying PSP is shown in Figure 1A. The crude polysaccharides extracted from PS had a carbohydrate content of 65.40% ± 2.19%. Subsequent decolorization and deproteinization processes reduced the protein content to 0.6003% ± 0.0071% (Table S1). To identify the most potent anti-inflammatory candidate for subsequent mechanistic research, four distinct polysaccharide fractions were isolated from the crude polysaccharides via ion-exchange chromatography, which were designated as PSP-1, PSP-2, PSP-3, and PSP-4, with respective yields of 20.72%, 22.68%, 35.4%, and 3.72% (Figure 1B). These polysaccharide fractions underwent further purification via a Sephadex G100 column, resulting in the sub-fractions of PSP-1a, PSP-1b, PSP-2a, and PSP-3a (Figure 1C–E).

### 3.2. Screening of PSP Fractions for Anti-Inflammatory Effects

**Figure 2:**
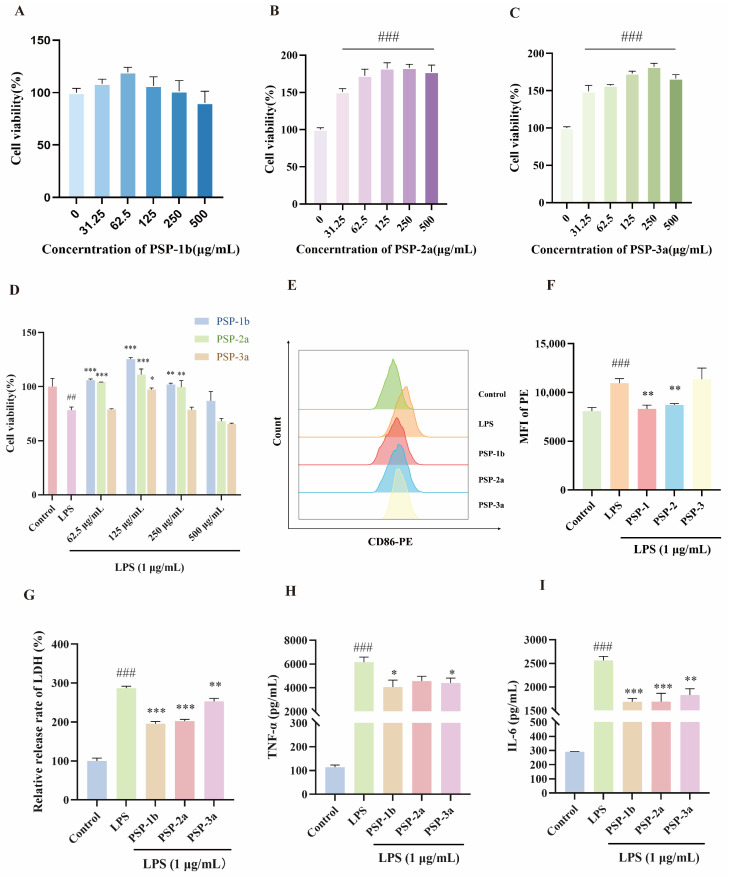
Cell viability assessment and screening of anti-inflammatory effects of PSP fractions. (**A**–**C**) RAW 264.7 cell viability assessment of PSP-1b (**A**), PSP-2a (**B**), and PSP-3a (**C**) via CCK8 assay n = 6). (**D**) Effect of PSP on the viability of RAW 264.7 cells after LPS (1 μg/mL) stimulation. (**E**,**F**) Effects of PSP-1b on polarization of macrophages (n = 3). (**G**–**I**) Effects of PSP-1b on the secretion of LDH (**G**), TNF-α (**H**), and IL-6 (**I**) from macrophages (n = 3). ^##^ *p* < 0.01, and ^###^ *p* < 0.001, compared to the control group; * *p* < 0.05, ** *p* < 0.01, and *** *p* < 0.001 compared to LPS group. Values are presented as mean ± SEM.

The effects of PSP-1b, PSP-2a, and PSP-3a on the viability of RAW 264.7 cells were first investigated. Results from the CCK8 assay indicated that all PSP fractions exhibited no significant toxicity to RAW 264.7 cells at concentrations ranging from 31.25 to 500 µg/mL. Furthermore, PSP-2a and PSP-3a were found to promote cell proliferation (Figure 2A–C). This is a common and well-documented property of many bioactive plant polysaccharides, often attributed to their ability to provide mild metabolic stimulation and nutritional support to cells in a steady state, which aligns with established literature [47]. Moreover, these PSP fractions protected cells from the impaired viability caused by LPS (Figure 2D). LPS stimulation has been reported to reduce macrophage activity via elevated LDH release and to induce pro-inflammatory cytokines that contribute to host damage [48,49]. Results indicated that PSP-1b, PSP-2a, and PSP-3a differentially alleviated the polarization of macrophages towards the M1 phenotype (Figure 2E,F), inhibited intracellular LDH release (Figure 2G), and reduced secretion of pro-inflammatory cytokines (IL-6 and TNF-α) (Figure 2H,I). Notably, PSP-1b exhibited a strong anti-inflammatory efficacy compared to other PSP fractions at 125 µg/mL. Collectively, PSP displayed significant anti-inflammatory properties, with PSP-1b demonstrating the strongest anti-inflammatory efficacy, making it a focus for subsequent molecular structural characterization and mechanistic explorations of anti-inflammatory activity.

### 3.3. Compositional Analysis of PSP-1b

**Figure 3:**
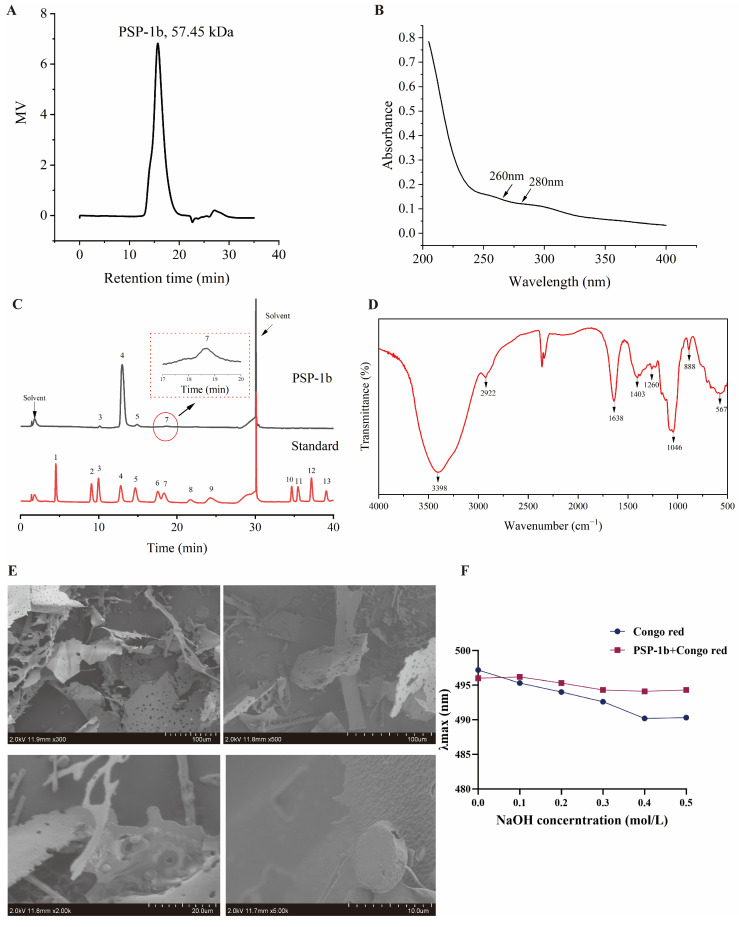
Compositional analysis of PSP-1b. (**A**) HPGPC chromatogram of PSP-1b. (**B**) UV–vis absorption spectrum of PSP-1b. (**C**) HPAEC chromatograms of monosaccharide standards and the PSP-1b sample. Peaks correspond to 1-Fuc, 2-Rha, 3-Ara, 4-Gal, 5-Glc, 6-Xyl, 7-Man, 8-Fru, 9-Rib, 10-GalA, 11-GulA, 12-GlcA, and 13-ManA. (**D**) FT-IR absorption spectrum of PSP-1b. (**E**) Analysis of the morphological properties of PSP-1b. (**F**) Plot of the maximum absorption of the PSP-1b–Congo red complex at varying concentrations of NaOH.

The results indicated that the molecular weight of PSP-1b was 57.45 kDa (Figure 3A). UV–vis spectroscopy revealed no absorption peaks at 260 nm and 280 nm, confirming that the sample contained no nucleic acid or protein components (Figure 3B). Furthermore, the carbohydrate content of PSP-1b was 93.99% ± 3.49%, while the protein content was 0.0170% ± 0.0006% (Table S1), suggesting that PSP-1b is a highly pure polysaccharide fraction.

Monosaccharide composition analysis indicated that PSP-1b consists of Ara, Gal, Glc, and Man, with molar ratios of Ara:Gal:Glc:Man = 1.49:94.78:2.24:1.49. No uronic acid components were detected (Figure 3C).

Infrared spectroscopy analysis revealed characteristic absorption bands of PSP-1b at key wavenumbers (Figure 3D). The peak observed at 3398.85 cm^−1^ is linked to -OH stretching vibrations found in the glycosidic ring [50], whereas the peak at 2922.45 cm^−1^ is associated with C-H stretching vibrations [51]. The notable signal observed at 1403.38 cm^−1^ arises from C-H bending vibrations in the pyranose rings [52]. A peak near 1638.54 cm^−1^ is attributed to -OH bending vibrations of water that has been absorbed [53], while the signal at 1260.21 cm^−1^ corresponds to C-H bending vibrations. Additionally, the peak at 1046.27 cm^−1^ is indicative of the C-O-C ether bond [54], and the signal at 888.94 cm^−1^ suggests the potential existence of β-configured pyranose [55].

### 3.4. Morphological Analysis

Scanning electron microscopy of PSP-1b at 300×, 500×, 2000×, and 5000× magnifications (Figure 3E) revealed irregular, fragment-like structures with fibrous branches and fine filaments. These features suggest that it may possess a high molecular weight and degree of branching, leading to tight interactions among the glyco-conjugate chains. At a magnification of 5000×, the surface of PSP-1b appeared rough with small papillary protrusions, indicating that PSP-1b has good solubility [56,57].

The Congo red assay was employed to identify the triple helical structure of PSP-1b. In alkaline solutions, Congo red can form a unique complex with the triple helical polysaccharide, resulting in a shift in the maximum absorption wavelength (λmax) as the concentration of NaOH increases [58]. As evidenced in Figure 3F, the λmax of the PSP-1b–Congo red complex exhibited negligible variations with increasing NaOH concentration, which demonstrates that PSP-1b does not adopt a triple helical conformation.

### 3.5. Structural Characterization of PSP-1b

To gain deeper insights into the molecular structure of PSP-1b, methylation analysis as well as 1D and 2D NMR spectroscopy were performed. The resulting PMAA derivatives were analyzed using GC–MS, and the total ion chromatogram (TIC) (Figure S2A) revealed three major peaks corresponding to distinct glycosidic linkages of PSP-1b, and the corresponding molar ratios are shown in Table 1. With reference to established literature [59,60,61], for PSP-1b, the three peaks in the TIC profile were identified as 1,5-di-O-acetyl-2,3,4,6-tetra-O-methyl galactitol (Figure S2B), 1,4,5-tri-O-acetyl-2,3,6-tri-O-methyl galactitol (Figure S2C), and 1,4,5,6-tetra-O-acetyl-2,3-di-O-methyl galactitol (Figure S2D). Subsequently, 1D and 2D NMR spectroscopy confirmed these structural features.

**Figure 4:**
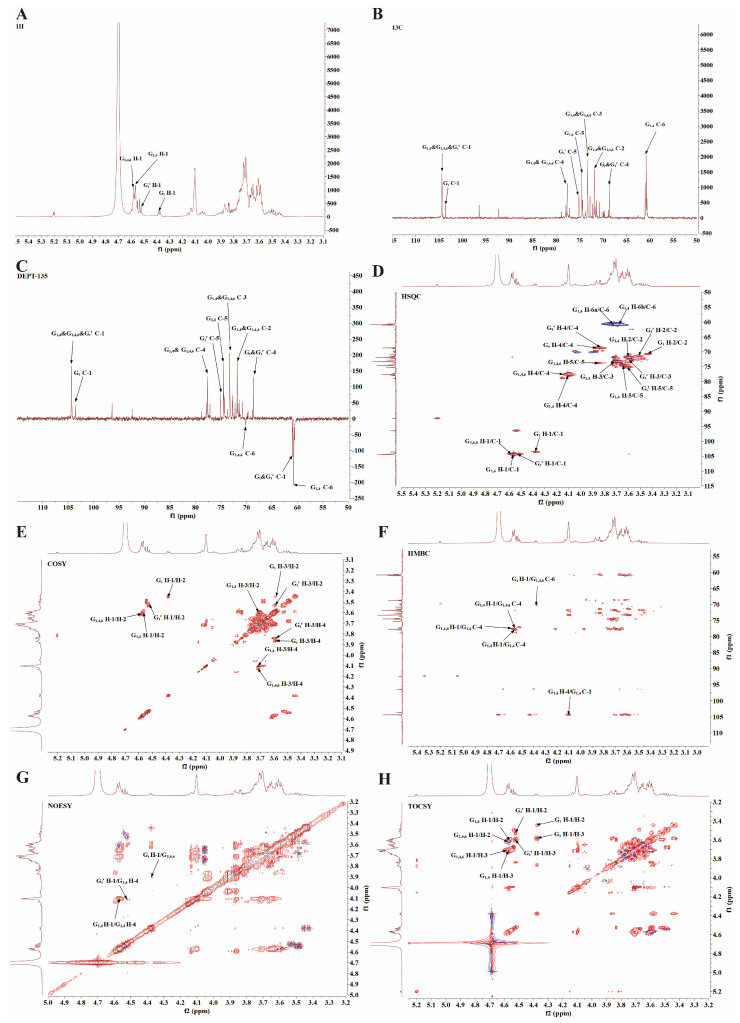
Structural characterization of PSP-1b. (**A**–**C**) NMR spectra: (**A**) ^1^H, (**B**) ^13^C, and (**C**) DEPT-135. (**D**–**H**) NMR spectra: (**D**) HSQC, (**E**) COSY, (**F**) HMBC, (**G**) NOESY, and (**H**) TOCSY.

The ^1^H NMR spectrum of PSP-1b (Figure 4A) exhibited characteristic polysaccharide signals between δ 3.0 and 5.5 ppm, with anomeric protons (H-1) resonating in the δ 4.4–5.5 ppm region. However, significant signal overlap in both the anomeric and aliphatic regions (δ 3.0–4.4 ppm) complicated the specific assignment of individual proton resonances [62]. In contrast, the ^13^C NMR spectrum (Figure 4B) exhibited improved resolution with anomeric carbons visible in the δ 90–110 ppm region. Additional signals between δ 65 and 85 ppm were attributed to ring carbons (C-2–C-5), while substituted and unsubstituted C-6 methylene carbons were identified near δ 69 ppm and δ 60 ppm, respectively.

Monosaccharide composition analysis revealed Gal as the predominant sugar, while methylation analysis indicated major residues of 1,4-Gal*p*, along with t-Gal*p* and 1,4,6-Gal*p*. These results suggested the presence of galactan structural units in PSP-1b [63]. Interpretation of ^1^H NMR, ^13^C NMR, DEPT-135, HSQC, COSY, HMBC, NOESY, and TOCSY spectra further revealed multiple distinct anomeric signals suitable for structural characterization (Figure 4A–H). All chemical shift assignments are compiled in Table 2. In the HSQC spectrum, residues G_1,4_ and G_1,4,6_ both exhibited characteristic anomeric signals within the typical β-Gal*p* region (δ_H_ 4.50–4.66/δ_C_ 99–105 ppm) [63]. For G_1,4_, the anomeric correlation at δ_H/C_ 4.56/104.33 ppm was fully assigned using COSY and HSQC spectra: H-1–H-6 at δ 4.56, 3.60, 3.71, 4.10, 3.64, and 3.67/3.73 ppm, and C-1–C-6 at δ 104.33, 71.73, 73.30, 77.60, 74.54, and 60.74 ppm. Glycosylation shifts at C-1 and C-4 confirmed its identity as →4)-β-D-Gal*p*-(1→ [64]. Similarly, the anomeric signal of G_1,4,6_ was assigned at δ_H/C_ 4.58/104.43 ppm, with proton and carbon resonances at δ 4.58, 3.61, 3.71, 4.13, 3.86, and 4.03/3.90 ppm for H-1–H-6, and δ 104.43, 71.84, 73.30, 77.60, 73.69, and 70.13 ppm for C-1–C-6. Downfield displacements of C-4 and C-6 confirmed glycosylation at both positions, consistent with the structure →4,6)-β-D-Gal*p*-(1→ [65,66,67].

Residue G_t_′ exhibited an anomeric correlation at δ_H/C_ 4.52/104.25 ppm in the HSQC spectrum, consistent with a β-galactopyranosyl unit. Full signal assignment revealed H-1–H-6 at δ 4.52, 3.52, 3.59, 3.83, 3.60, and 3.66/3.73 ppm and C-1–C-6 at δ 104.25, 71.35, 72.71, 68.67, 75.15, and 60.99 ppm, supporting its identification as the disaccharide segment β-D-Gal*p*-(1→4)-β-D-Gal*p*-(1→ [64]. Similarly, residue G_t_ showed an anomeric signal at δ_H/C_ 4.38/103.57 ppm, with H-1–H-6 at δ 4.38, 3.45, 3.58, 3.87, 3.81, and 3.66/3.73 ppm and C-1–C-6 at δ 103.57, 70.74, 71.84, 68.67, 73.69, and 60.99 ppm. Glycosylation shifts at C-4 and C-6 indicated a β-D-Gal*p*-(1→4,6)-β-D-Gal*p*-(1→ motif [64,68].

**Figure 5:**
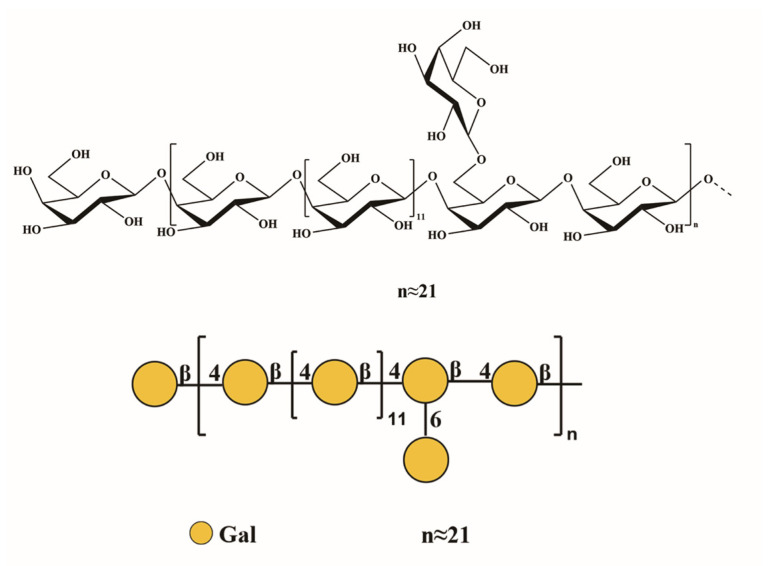
Proposed chemical structure of PSP-1b.

Complete proton and carbon resonance assignments of the major glycosyl residues were achieved through integrated analysis of 2D NMR (COSY, HSQC, HMBC, NOESY, and TOCSY) together with 1D ^1^H NMR, ^13^C NMR, DEPT-135, monosaccharide composition, and methylation data. Chemical shifts were further validated against literature values for similarly substituted sugar residues. The glycosidic linkage sequence was elucidated using HMBC (Figure 4F) and NOESY (Figure 4G) spectra. Key HMBC correlations revealed inter-residue connectivities via long-range couplings between anomeric protons and aglycone carbons, while NOESY provided complementary evidence through spatial proximities between protons across glycosidic bonds. An HMBC correlation between H-1 (δ 4.56) of residue G_1,4_ and C-4 (δ 77.60) of the same residue, along with a corresponding H-4 (δ 4.10)/C-1 (δ 104.33) correlation, was supported by a NOESY cross-peak between H-1 and H-4 of G_1,4_, indicating a →4)-β-D-Gal*p*-(1→4)-β-D-Gal*p*-(1→ linkage [68]. Additional HMBC correlations between H-1 of G_1,4_ (δ 4.56) and C-4 of G_1,4,6_ (δ 77.60) indicated a →4)-β-D-Gal*p*-(1→4,6)-β-D-Gal*p*-(1→ linkage, while a correlation between H-1 of G_1,4,6_ (δ 4.58) and C-4 of G_1,4_ (δ 77.60) suggested a →4,6)-β-D-Gal*p*-(1→4)-β-D-Gal*p*-(1→ connection. Furthermore, an HMBC correlation between H-1 of G_t_ (δ 4.38) and C-6 of G_1,4,6_ (δ 70.13), supported by a NOESY cross-peak between H-1 of G_t_ and H-6 of G_1,4,6_ (δ 3.90), confirmed a β-D-Gal*p*-(1→6) linkage. A NOESY cross-peak between H-1 of G_t_′ (δ 4.52) and H-4 of G_1,4_ (δ 4.10) indicated a β-D-Gal*p*-(1→4)-β-D-Gal*p*-(1→ linkage. Methylation analysis revealed a molar ratio of approximately 13:1 for →4)-β-D-Gal*p*-(1→ to →4,6)-β-D-Gal*p*-(1→ residues. Integrated with monosaccharide composition and NMR data, there results indicate that the primary structural feature is a galactan backbone composed of →4)-β-D-Gal*p*-(1→ units, partially substituted at O-6 by side chains predominantly terminated with β-D-Galp-(1→ residues. A proposed repeating unit of this structure is depicted in Figure 5.

### 3.6. PSP Alleviates DSS-Induced UC in Mice

**Figure 6:**
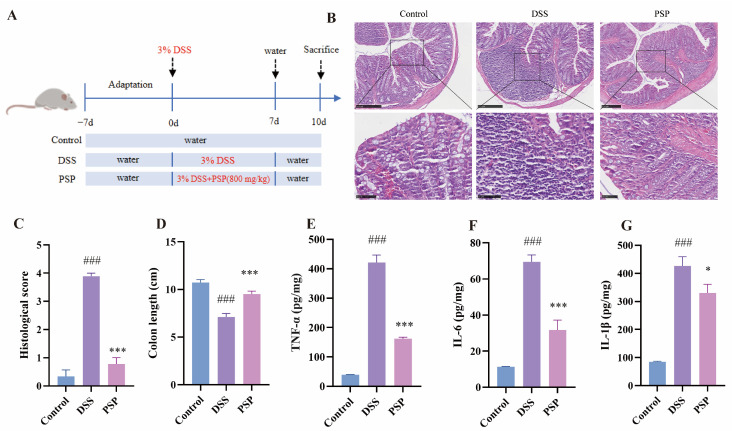
PSP alleviates DSS-induced colitis symptoms in mice. (**A**) Experimental design schematic. (**B**) H&E staining of colon tissues from different groups. Scale bar: 250 μm (n = 3). (**C**) Histological scores (n = 3). (**D**) Quantification of colon length in different groups (n = 6). (**E**–**G**) Levels of inflammatory cytokines in mouse colon tissues (n = 6). ^###^ *p* < 0.001, compared to the control group; * *p* < 0.05 and *** *p* < 0.001, compared to the DSS group. Values are presented as mean ± SEM.

The establishment of the acute UC model and the corresponding drug intervention protocol are illustrated in Figure 6A. To assess the role of PSP against DSS-induced colonic injury, colon length and histopathological analysis were measured. As expected, mice in the DSS group exhibited a significant reduction in colon length, which was effectively restored by PSP treatment. H&E staining further revealed that DSS challenge caused severe crypt destruction, mucosal erosion, and extensive infiltration of inflammatory cells. In contrast, these pathological damages were notably alleviated in the PSP-treated group, indicating that PSP exerts a potent protective effect against UC in vivo (Figure 6B–D).

Given the critical role of inflammatory cytokines in intestinal inflammation [69], we next measured the levels of TNF-α, IL-1β, and IL-6 in colonic tissues. Consistent with the severe tissue damage, the DSS group exhibited a significant increase in the secretion of these inflammatory cytokines. In contrast, PSP treatment significantly suppressed their secretion (Figure 6E–G), indicating that PSP can significantly mitigate the inflammatory response under UC conditions.

### 3.7. PSP Inhibits NET–Macrophage Crosstalk in the Colons of UC Mice

**Figure 7:**
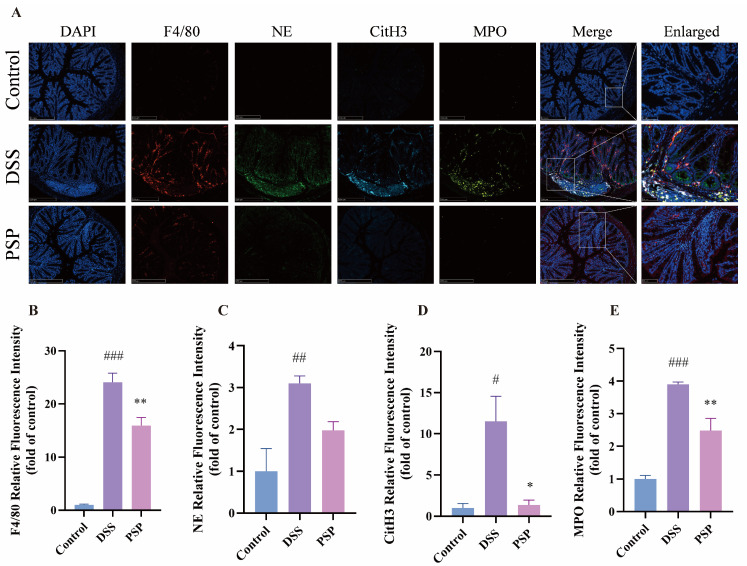
PSP alleviates the NET–macrophage crosstalk in UC mice. (**A**) Representative images showing F4/80, NE, CitH3, and MPO expression and co-localization in different groups (n = 3). Scale bars: 250 μm (**left**) and 50 μm (**right**). (**B**–**E**) Quantitative analysis of F4/80, NE, CitH3, and MPO expression (n = 3). ^#^ *p* < 0.05, ^##^ *p* < 0.01, and ^###^ *p* < 0.001, compared to the control group; * *p* < 0.05 and ** *p* < 0.01, compared to the DSS group. Values are presented as mean ± SEM.

As key innate immune cells in the gut, macrophages are often recruited to inflammatory sites in UC, where their abnormal infiltration can drive persistent mucosal damage and sustain a cycle of inflammation [70]. Concurrently, NET deposition is a hallmark of UC progression. Therefore, to evaluate the impact of PSP on macrophage infiltration and NET deposition in the UC context, we measured the expression of the macrophage marker F4/80 and key NET components (NE, MPO, and CitH3) in colonic tissues. Immunofluorescence analysis revealed that DSS challenge significantly increased the levels of NE, MPO, and CitH3 compared to the control group, with these markers notably co-localizing around macrophages. In contrast, PSP treatment markedly reduced the expression of all these NET markers (Figure 7A–E), indicating that its therapeutic effect against UC involves suppression of NET formation and NET–macrophage crosstalk.

### 3.8. PSP-1b Inhibits the Levels of Inflammatory Mediators in RAW 264.7 Cells Induced by LPS and NETs

**Figure 8:**
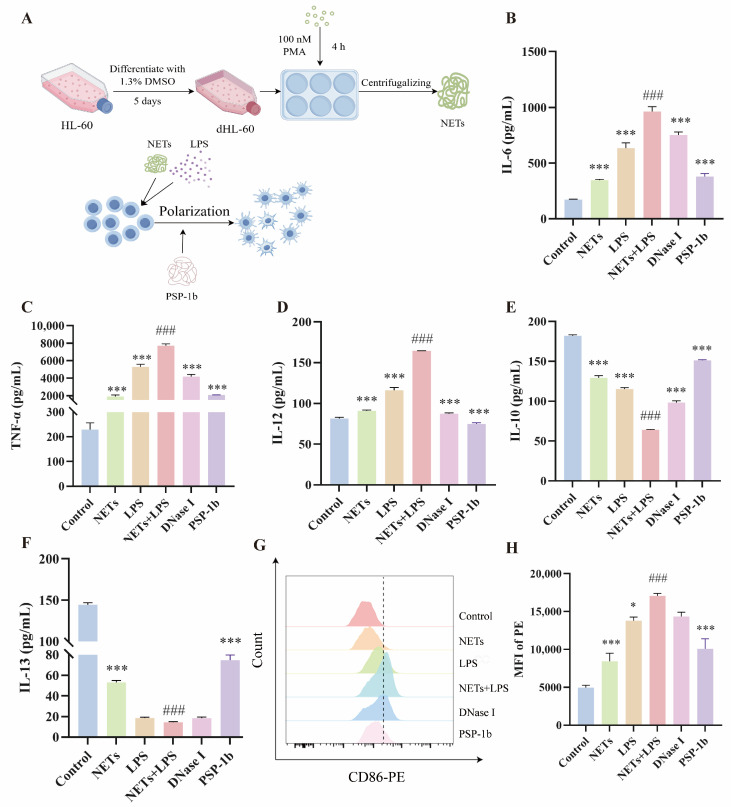
PSP-1b inhibits the levels of inflammatory mediators and M1-phenotype polarization in RAW 264.7 cells induced by NETs and LPS. (**A**) Cell experiment design flow chart. HL-60 cells were differentiated into dHL-60 cells using 1.3% DMSO and PMA stimulation to form NETs. These NETs were co-stimulated with LPS in RAW 264.7 cells to induce inflammation. PSP-1b was added to explore the anti-inflammatory efficacy. (**B**–**F**) Effects of PSP-1b on the secretion of (**B**) IL-6, (**C**) TNF-α, (**D**) IL-12, (**E**) IL-10, and (**F**) IL-13 from RAW 264.7 cells (n = 6). (**G**,**H**) Effects of PSP-1b on polarization of macrophages (n = 3). ^###^ *p* < 0.001, compared to the control group; * *p* < 0.05 and *** *p* < 0.001, compared to the NETs + LPS group. Values are presented as mean ± SEM.

To evaluate the anti-inflammatory activity of PSP-1b, we first differentiated HL-60 cells into neutrophil-like dHL-60 cells, and the formation of NETs was induced. The isolated NETs were then used to stimulate RAW264.7 macrophages (Figure 8A). We measured the levels of IL-6, IL-12 (p70), and TNF-α as well as the anti-inflammatory cytokines (IL-10 and IL-13) in RAW 264.7 cells. As shown in Figure 8B–F, the unstimulated control group exhibited baseline levels of inflammatory cytokines, while the group stimulated with NETs and LPS demonstrated significant upregulation of pro-inflammatory cytokines alongside a marked decrease in anti-inflammatory cytokines, confirming the reactivity of this cellular model to inflammatory stimuli. Compared to the model group, digestion of NETs with DNase I attenuated inflammatory responses, reducing pro-inflammatory cytokine secretion and elevating anti-inflammatory cytokine levels. Notably, PSP-1b treatment significantly reduced inflammation levels, substantially decreasing pro-inflammatory cytokines while markedly increasing anti-inflammatory cytokines, suggesting a superior anti-inflammatory effect of PSP-1b. Additionally, our findings indicated that the pro-inflammatory effects of NETs may be associated with their structural integrity.

### 3.9. PSP-1b Inhibited the M1-Phenotype Polarization of RAW 264.7 Cells

Macrophages could be activated into M1-phenotype and M2-phenotype macrophages by various factors in response to an inflammatory response. Modulating the activation state of macrophages to improve the inflammatory environment is an effective strategy for disease treatment [71,72]. Flow cytometric analysis revealed that stimulation with LPS and NETs significantly increased the population of CD86-positive macrophages and promoted M1-phenotype polarization, confirming successful inflammatory model establishment. Furthermore, degradation of NETs by DNase I attenuated this M1 polarization, suggesting that the pro-inflammatory activity of NETs may depend on their structural integrity rather than hydrolytic fragments. Crucially, PSP-1b administration in the PSP-1b + NETs + LPS group notably decreased both the CD86-positive cell population and M1-phenotype macrophages, demonstrating that PSP-1b exerts potent anti-inflammatory effects in mitigating inflammatory responses (Figure 8G,H).

### 3.10. PSP-1b Inhibits TLR4/MAPK/NF-κB Signaling Pathway

**Figure 9:**
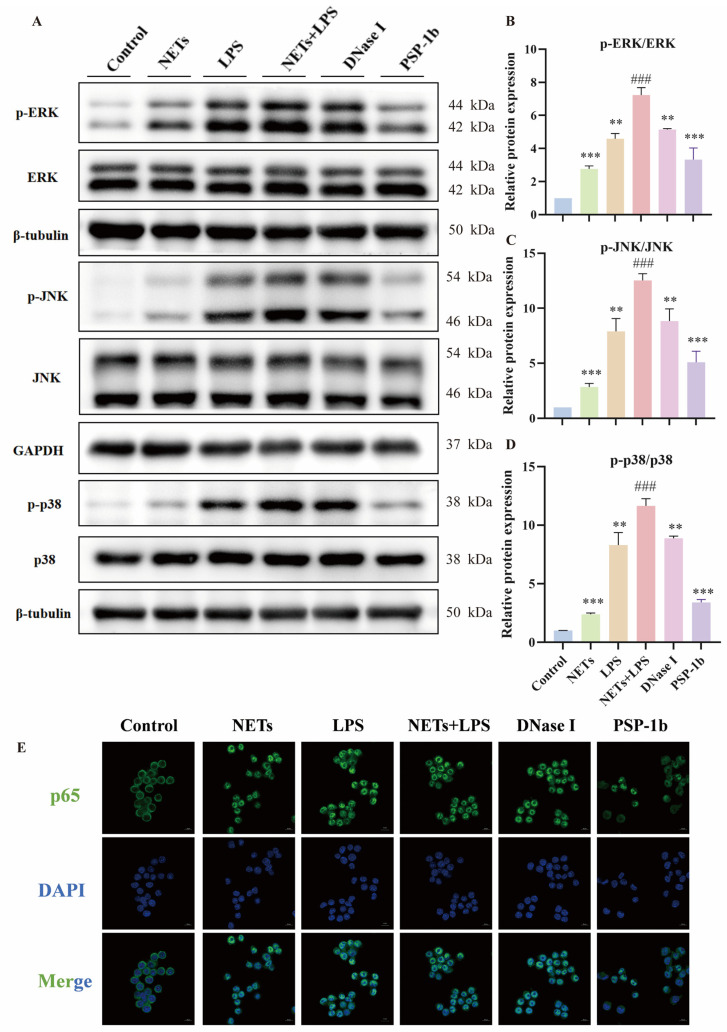
PSP-1b inhibits the TLR4/MAPK/NF-κB signaling pathway. (**A**–**D**) Quantitative analysis of the phosphorylation levels of ERK, JNK, and p38 based on the blotting images. (**E**) Representative immunofluorescent images showing NF-κB p65 nuclear translocation in RAW 264.7 cells. ^###^ *p* < 0.001 compared to the control group; ** *p* < 0.01 and *** *p* < 0.001 compared to the NETs + LPS group. Values are presented as mean ± SEM.

Substantial evidence establishes that the development of inflammation is mechanistically linked to phosphorylation-mediated activation of the MAPK/NF-κB signaling pathway [73,74]. The above experiments demonstrated that PSP-1b significantly reduced LPS/NET-induced inflammation by reducing M1-phenotype cytokines (TNF-α and IL-6) and inhibiting M1 polarization of macrophages. To investigate whether this effect involves the MAPK signaling pathway, we assessed the phosphorylation of key pathway components (p38, JNK, and ERK) via Western blotting. As shown in Figure 9A–D, LPS/NET co-stimulation significantly increased the phosphorylation of p38, JNK, and ERK without altering total protein levels, indicating that the MAPK signaling pathway was activated. PSP-1b treatment markedly suppressed this phosphorylation signature compared to the LPS/NET co-stimulation group, suggesting that PSP-1b could significantly inhibit the activation of the MAPK signaling pathway, thereby preventing M1 polarization of macrophages and reducing the release levels of inflammatory factors, exerting an anti-inflammatory effect.

Furthermore, nuclear translocation of p65 was monitored to assess the anti-inflammatory mechanism of PSP-1b (Figure 9E). Immunofluorescence analysis revealed predominant cytoplasmic p65 localization in unstimulated controls. However, LPS/NETs co-stimulation markedly enhanced nuclear iFluo^TM^ 488-labeled p65 signal intensity. In contrast, PSP-1b treatment significantly attenuated nuclear accumulation while increasing cytoplasmic retention. This indicates that PSP-1b could effectively inhibit p65 nuclear translocation, suppressing NF-κB-mediated inflammatory activation.

### 3.11. PSP-1b Directly Binds TLR4

**Figure 10:**
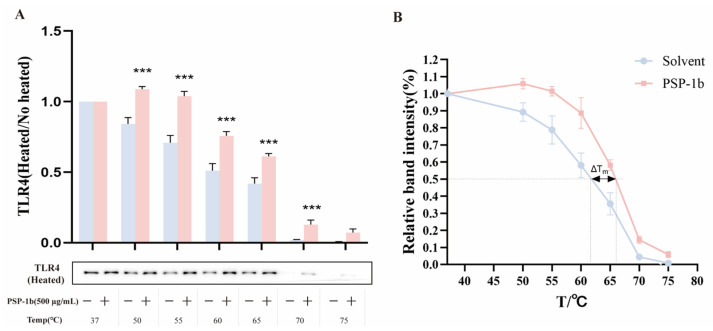
Cellular thermal shift assay (CETSA) of PSP-1b with RAW 264.7 cell lysates. (**A**) Quantification of TLR4 shown in the panel. (**B**) PSP-1b binding increased TLR4 thermal stability (ΔTm = 2.72 °C). *** *p* < 0.001 compared to the negative control group (without PSP-1b). Values are presented as mean ± SEM.

Given that TLR4 is a membrane surface receptor responsible for activating the NF-κB and MAPK signaling pathways, we investigated whether PSP-1b directly binds to TLR4 and inhibits the consequent pathways. CETSA revealed that PSP-1b significantly enhanced thermal stability of TLR4 in RAW 264.7 cell lysates at temperatures ranging from 50 to 75 °C, with a ΔTm of 2.72 °C compared to the control group (Figure 10A,B). These findings indicate a direct interaction between PSP-1b and TLR4.

## 4. Discussion

Excessive formation of NETs can be recognized as damage-associated molecular patterns by pattern recognition receptors on the surface of immune cells, especially macrophages. This interaction activates macrophages through the TLR4/NF-κB signaling pathway, leading to the production of pro-inflammatory cytokines and the formation of a positive feedback loop. This ultimately triggers an inflammatory storm, thereby further exacerbating inflammation [75]. In UC, significant infiltration of neutrophils and increased levels of NETs are characteristic features believed to sustain persistent mucosal inflammation. Thus, targeting NET–macrophage crosstalk has become a potentially effective therapeutic approach for UC. In recent years, natural products have attracted considerable interest due to their anti-inflammatory and immunomodulatory activities. A growing number of studies have demonstrated that PS-derived polysaccharides can alleviate UC symptoms, enhance intestinal barrier function, and suppress excessive immune responses [76].

**Figure 11:**
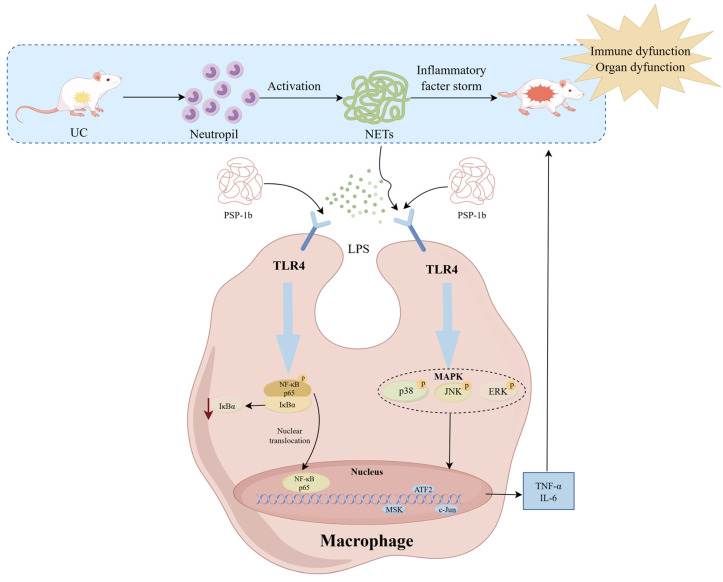
Schematic diagram depicting the mechanisms through which PSP-1b inhibits LPS/NET-induced inflammation via downregulation of the TLR4/MAPK/NF-κB signaling pathway.

In this study, we first demonstrated that PSP effectively alleviated DSS-induced UC symptoms while inhibiting both macrophage infiltration and NET formation. The observed co-localization of macrophage markers and NET markers in the DSS group implied a functional interplay during UC progression, which was abolished by PSP. To further investigate the mechanism through which PSP exerts its anti-UC effects via mitigation of NET–macrophage crosstalk, we isolated subfractions from PSP and identified PSP-1b as the most active component. PSP-1b exhibited the most potent anti-inflammatory effects among all fractions, significantly reducing inflammatory cytokines release, LDH activity, and reversing M1 macrophages polarization. PSP-1b was characterized as a galactan with a molecular weight of approximately 57.45 kDa, featuring a backbone primarily consisted of →4)-β-D-Gal*p*-(1→ residues with occasional O-6 branching, predominantly terminated by β-D-Gal*p*-(1→ side chains. Further mechanistic investigations revealed that PSP-1b suppresses activation of the MAPK/NF-κB signaling pathway by targeting TLR4, thereby effectively reversing LPS- and NET-induced M1 polarization of macrophages. This action significantly reduced the levels of pro-inflammatory cytokines while facilitating the release of anti-inflammatory cytokines. Collectively, PSP-1b disrupts the inflammatory amplification cascade driven by NET–macrophage crosstalk, ultimately attenuating the pathological progression of UC (Figure 11).

Polysaccharide bioactivity is closely linked to structural characteristics, particularly molecular weight [77]. Consistent with previous reports that low-molecular-weight polysaccharides often exhibit enhanced immunomodulatory capacity [78], PSP-1b (57.45 kDa) demonstrated superior anti-inflammatory activity compared to higher molecular weight fractions (PSP-2a, 123.7 kDa; PSP-3a, 101.7 kDa) (Figure S1), more effectively suppressing inflammatory cytokine release and attenuating M1 macrophage polarization. Furthermore, the molecular weight range of polysaccharides determines their mechanism of action. Low-Mw polysaccharides (<10 kDa) can cross cellular membrane barriers and exhibit enhanced immunostimulatory properties [79], whereas medium- (10–1000 kDa) and high-Mw (>1000 kDa) polysaccharides primarily bind to cell surface receptors to exert immunomodulatory and antitumor effects [80]. Notably, PSP-1b falls within the medium-molecular-weight range (10–100 kDa), which has been strongly correlated with TLR4 binding affinity. Monosaccharide composition further dictates functional specificity and receptor recognition [81]. PSP-1b is primarily composed of galactose. Previous studies have isolated various galactans from steamed PS, such as a 1,4-β-D-galactan with a molecular weight of 42 kDa and a polymerization degree of 6 [82], another 1,4-β-D-galactan of 7019 Da with a polymerization degree of 8 [83], and a galactan of 14.4 kDa with a polymerization degree of 13 [76]. These galactans have demonstrated promising immunomodulatory or gut microbiota-regulating activities, which are consistent with the findings of this study. Additionally, macrophage surface receptors, including TLR4, mannose receptor, galectin, and Dectin-1, are capable of recognizing specific saccharide motifs such as mannose-, galactose-, or β-glucan-rich structures, thereby triggering downstream signaling pathways and modulating macrophage function [84]. Notably, polysaccharides capable of binding TLR4 frequently contain characteristic monosaccharides such as Gal, Glc, Man, Ara, and Rha [81]. The galactose-rich composition of PSP-1b thus supports a mechanism involving TLR4 recognition, contributing to its observed anti-inflammatory activity.

LPS translocation and subsequent NET deposition are key events in UC pathology and are thought to sustain a mutual pro-inflammatory cycle. To mechanistically unravel the contribution of NET–macrophage crosstalk within this cycle, the present study established an in vitro model to simulate the intestinal microenvironment of UC, characterized by bacterial leakage and NET deposition. This was achieved by inducing neutrophil-like differentiation of HL-60 cells (dHL-60), stimulating NETosis, and subsequently co-stimulating RAW264.7 macrophages with isolated NETs and LPS. Notably, both LPS and NETs individually activated the MAPK and NF-κB signaling pathways, with the most pronounced activation occurring under co-stimulation. These results confirm the successful establishment of a cellular model that recapitulates the dysregulated intestinal microenvironment and NET deposition observed in UC. In this model, macrophages polarized towards the M1 phenotype, accompanied by significantly elevated levels of pro-inflammatory cytokines such as IL-6 and TNF-α, indicating that NETs engage in crosstalk with macrophages to regulate their polarization state, thereby exacerbating the progression of UC. Treatment of NETs with DNase I attenuated their pro-inflammatory effects, consistent with previously reported findings [85] and clinical therapeutic strategies [86]. By degrading the DNA scaffold of NETs, DNase I facilitates the dispersal of cytotoxic components (e.g., NE and cathepsin G) as well as pro-inflammatory molecules (e.g., citrullinated histone H3 and HMGB1) into the extracellular environment [87]. This dissolution abrogates their locally concentrated cytotoxic and inflammatory impacts. These results further support that targeted disruption of detrimental NET–macrophage crosstalk represents a promising strategy for mitigating the progression of UC.

Macrophage-driven immune dysregulation in the intestinal microenvironment plays a critical role in the pathogenesis of UC. Macrophages exhibit high plasticity, with the M1 subtype contributing to early inflammatory immune responses [88]. Flow cytometry analysis revealed that PSP-1b treatment significantly reduced the proportion of CD86+ macrophages, a key M1 phenotype surface marker, indicating effective suppression of LPS/NET-induced M1 polarization. This attenuation in polarization subsequently alleviates inflammatory responses, suggesting a potential therapeutic benefit of PSP-1b in mitigating the pathological progression of UC. Furthermore, elevated levels of pro-inflammatory mediators such as IL-1β, IL-6, and TNF-α are closely associated with the pathogenesis of UC and disease severity [89]. The present study demonstrated that PSP-1b significantly reduced the production of pro-inflammatory cytokines, including TNF-α, IL-12, and IL-6, while promoting the secretion of anti-inflammatory cytokines such as IL-10 and IL-13. This shift effectively restores the balance between pro- and anti-inflammatory cytokines, underscoring the substantial anti-inflammatory effect of PSP-1b in the context of UC and its therapeutic potential in mitigating disease progression.

The MAPK/NF-κB signaling pathway serves as a critical hub regulating the transcription of inflammation-associated genes, and its phosphorylation level is positively correlated with the severity of inflammation, playing a pivotal role in the development of UC [90]. In this study, PSP-1b significantly suppressed the phosphorylation of the MAPK pathway compared to the model group, suggesting its potent efficacy in alleviating UC-related inflammation in vitro. Under resting conditions, NF-κB remains sequestered in the cytoplasm by IκB proteins; upon inflammatory stimulation, IκB phosphorylation and degradation enable NF-κB nuclear translocation, where it drives the expression of various pro-inflammatory cytokines and mediators. This mechanism is closely associated with the pathogenesis of UC [91]. Immunofluorescence analysis revealed that PSP-1b treatment significantly inhibited nuclear translocation of the p65 subunit, thereby blocking NF-κB activation and contributing to the alleviation of UC progression. TLR4, a pattern recognition receptor for pathogen-associated molecular patterns (PAMPs), activates downstream MAPK/NF-κB signaling pathways. To further validate the direct interaction between PSP-1b and TLR4, CETSA was performed. The results show that PSP-1b enhanced the thermal stability of TLR4, which is consistent with its structural features. These findings suggest that PSP-1b may compete with LPS and NETs for TLR4 binding sites, thereby disrupting NET–macrophage crosstalk. Consequently, by targeting TLR4, PSP-1b inhibits MAPK/NF-κB pathway activation, reduces pro-inflammatory cytokine secretion, restores cytokine balance, reverses M1 macrophage polarization, and ultimately attenuates the progression of UC.

Compared to conventional therapeutics, PSP-1b demonstrates distinctive advantages in both immunomodulatory capacity and digestive stability [92]. As a macromolecular compound, PSP-1b is poorly absorbed into the systemic circulation following oral administration. This property enables its high-concentration accumulation in the colonic lesions of UC, facilitating prolonged and concentrated interactions with macrophages recruited to inflammatory sites, thereby exerting sustained anti-inflammatory effects. Current NET-targeting therapeutic strategies face notable limitations. DNase I, though effective in digesting NETs’ DNA framework, can promote the release of cytotoxic factors such as proteases and histones, potentially aggravating tissue damage. Excessive NET degradation in the early phase of inflammation may also impair innate immunity and increase infection [93]. Furthermore, PAD4 inhibitors (including GSK484, Cl-amidine, and icariin), which suppress NET formation by inhibiting histone citrullination, are ineffective against preformed NETs and may cause dose-dependent adverse effects such as neuromuscular dysfunction and peripheral neuropathy [94]. Therefore, targeted disruption of the NET–macrophage crosstalk represents a promising therapeutic strategy. In this context, it is worthwhile to further investigate whether PSP-1b could be utilized in combination with other classes of therapeutic agents. Specifically, this approach involves using PAD4 inhibitors to suppress NET formation, DNase I to degrade pre-existing NETs, and PSP-1b to inhibit TLR4-mediated NET–macrophage crosstalk. Together, these agents form a complementary mechanism, which acts through a “cocktail” therapeutic strategy, to overcome the limitations of monotherapy, reduce individual drug dosages, minimize adverse effects, and potentially establish a novel direction for future treatment. Moreover, in the development and progression of UC, NETs interact with diverse immune cells that extend beyond macrophages, including T cells [95] and epithelial cells [96], and should not be overlooked. Further research is needed to determine whether PSP-1b modulates these additional interactions, broadening its potential immunoregulatory role in UC.

## 5. Conclusions

This study demonstrates that PSP protects against DSS-induced UC in mice by modulating the crosstalk between NETs and macrophages. Bioactivity-guided fractionation identified PSP-1b as the most promising anti-inflammatory subfraction. Comprehensive structural analysis characterized PSP-1b as a neutral heteropolysaccharide (57.45 kDa) with a backbone composed of →4)-β-D-Gal*p*-(1→ residues, and partially substituted at O-6 with side chains primarily terminating in β-D-Gal*p*-(1→. In vitro experiments further demonstrated PSP-1b’s capacity to attenuate LPS/NET-induced macrophage inflammation and M1 polarization by directly binding to the TLR4 receptor and inhibiting the downstream MAPK/NF-κB signaling pathway. We further established that the pro-inflammatory activity of NETs exhibits critical dependence on structural integrity. These findings underscore PSP-1b as a promising TLR4-targeted therapeutic candidate or functional food ingredient for inflammatory diseases, acting through disruption of the NET–macrophage axis. While this study establishes PSP-1b as a promising TLR4-targeted therapeutic candidate, the precise mechanisms through which it modulates NET interactions with other immune cells warrant further investigation. Moreover, future studies exploring PSP-1b in combination with PAD4 inhibitors or DNase I as part of a multi-targeted “cocktail” therapeutic strategy may further enhance its clinical potential against UC.

## Figures and Tables

**Table 1:** Methylation analysis of PSP-1b.

Linkage Types	Methylated Sugar	Characteristic Ions (*m*/*z*)
t-Gal*p*	1,5-di-O-acetyl-2,3,4,6-tetra-O-methyl galactitol	43.0, 60.0, 71.0, 86.9, 101.9, 118.0, 128.9, 144.9, 160.9, 173.0, 205.0
4-Gal*p*	1,4,5-tri-O-acetyl-2,3,6-tri-O-methyl galactitol	43.1, 60.0, 75.0, 86.9, 98.9, 118.0, 131.0, 142.9, 162.0, 173.0, 203.0, 233.0
4,6-Gal*p*	1,4,5,6-tetra-O-acetyl-2,3-di-O-methyl galactitol	44.0, 60.0, 74.0, 84.9, 101.9, 118.0, 126.9, 141.9, 128.9, 187.0, 201.0

**Table 2:** ^13^C and ^1^H NMR chemical shifts (ppm) of PSP-1b.

Residue	Glycosidic Linkage	Chemical Shift δ _H/C_ (ppm)						
			1	2	3	4	5	6a/6b
G_1,4_	→4)-β-D-Gal*p*-(1→	H	4.56	3.60	3.71	4.10	3.64	3.67/3.73
		C	104.33	71.73	73.30	77.6	74.54	60.74
G_1,4,6_	→4,6)-β-D-Gal*p*-(1→	H	4.58	3.61	3.71	4.13	3.86	4.03/3.90
		C	104.43	71.84	73.30	77.6	73.69	70.13
G_t_′	β-D-Gal*p*-(1→	H	4.52	3.52	3.59	3.83	3.60	3.66/3.73
		C	104.25	71.35	72.71	68.67	75.15	60.99
G_t_	β-D-Ga*lp*-(1→	H	4.38	3.45	3.58	3.87	3.81	3.66/3.73
		C	103.57	70.74	71.84	68.67	73.69	60.99

## Data Availability

The original contributions presented in this study are included in the article/Supplementary Material. Further inquiries can be directed to the corresponding author.

## Supplementary Materials

The following supporting information can be downloaded at https://www.mdpi.com/article/10.3390/nu18071046/s1. Figure S1: (A) HPGPC chromatogram of PSP-2a. (B) HPGPC chromatogram of PSP-3a. Figure S2: Total ion chromatogram of methylation products of PSP-1b (A) and mass spectra for corresponding peaks (B–D). Table S1:Composition of PSP and PSP-1b. Table S2: Colonic histopathological scoring method.
