# Supplementary material for: Structural Characterization and Anti-Colitis Mechanisms of Polygonatum sibiricum Polysaccharides via Modulation of Neutrophil Extracellular Traps (NETs)—Macrophage Crosstalk
Source: Nutrients. 2026 Mar 25;18(7):1046. doi: 10.3390/nu18071046 (PMC13074318; doi:10.3390/nu18071046)
Supplement: Supplementary file 1 [file nutrients-18-01046-s001.zip › nutrients-4198990-supplementary.pdf]

**Table S1.** Contents of PSP and PSP-1b

| | Carbohydrate content (%) | Protein content (%) |
|---|---|---|
| PSP | 65.40 ± 2.19 | 0.6003 ± 0.0071 |
| PSP-1b | 93.99 ± 3.49 | 0.0170 ± 0.0006 |

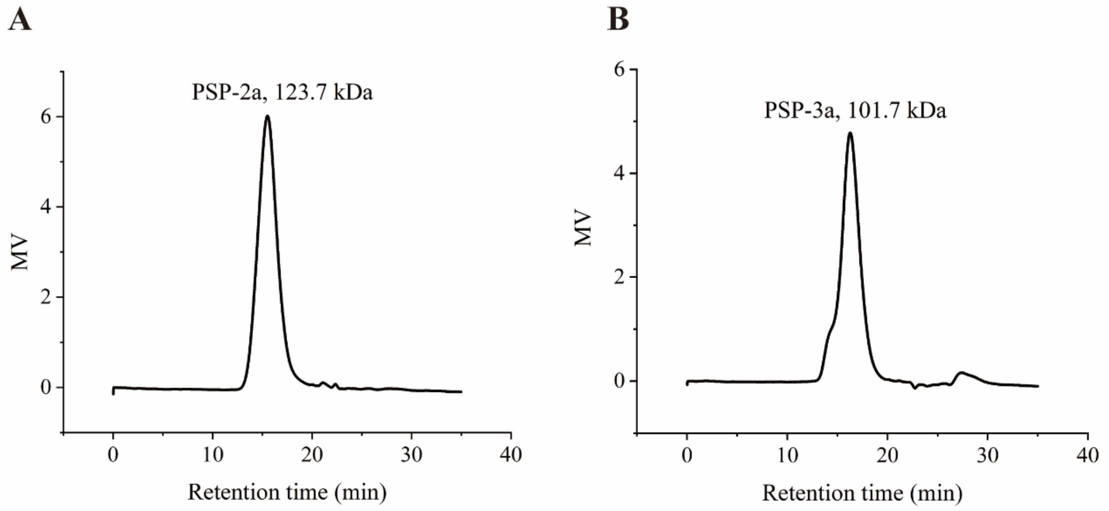


**Figure S1**. (A) The HPGPC chromatogram of PSP-2a. (B) The HPGPC chromatogram of PSP-3a.

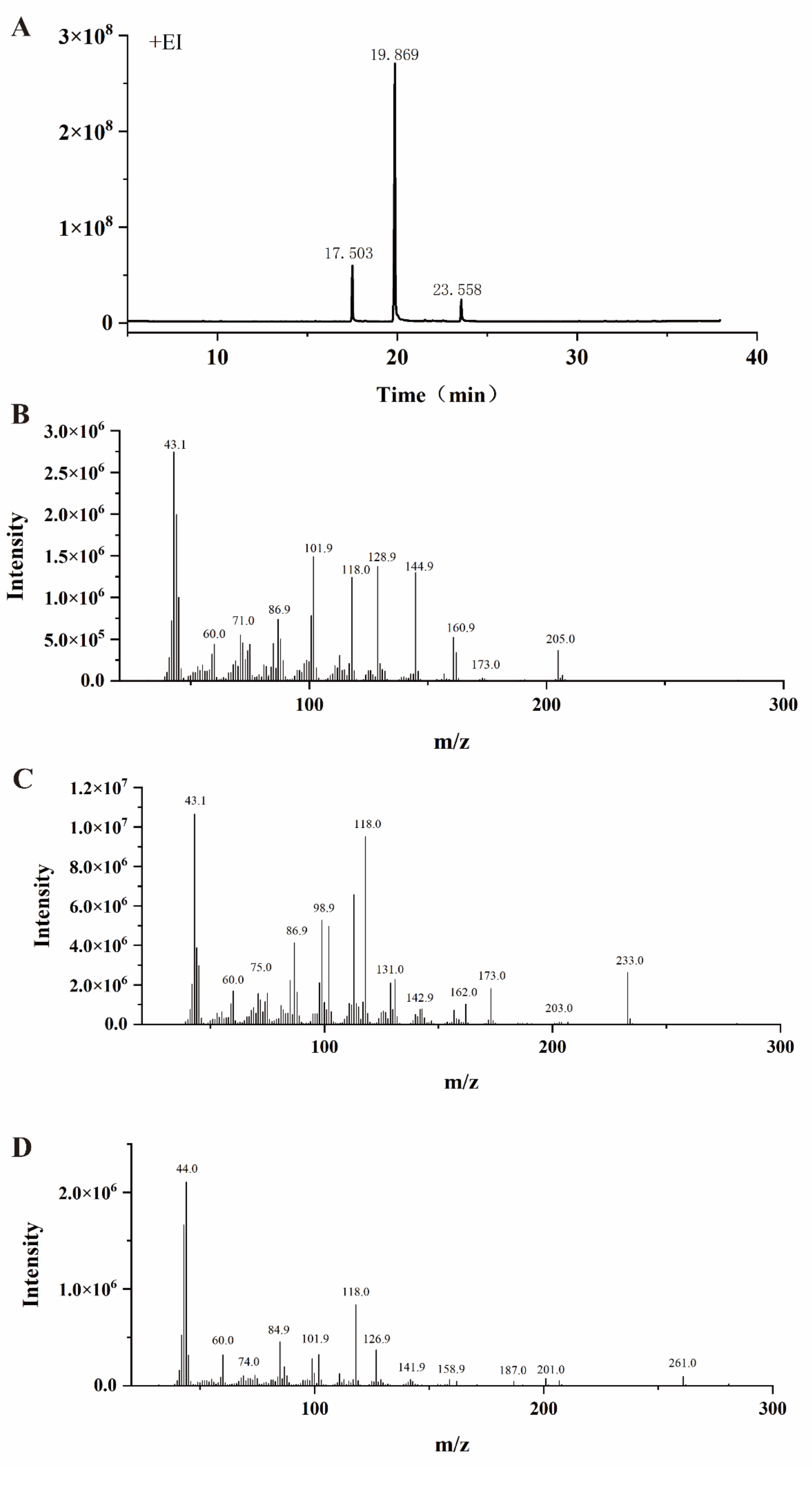


**Figure S2.** Total ion chromatogram for methylation products of PSP-1b (A) and mass spectra for corresponding peaks (B-D).

**Table S2.** Colonic histopathological scoring method

| **Mucosa ulcer** | **Crypt damage** | **Inflammatory cell infiltration** | **Inflammatory exudate** | **Fibrosis** | **Lesion range** | **Score** |
|---|---|---|---|---|---|---|
| None | None | None | None | None | <1% | 0 |
| Superficial erosion | Destruction of 1/3 of the basal crypts | Mild | Mild | Mild | 1-25% | 1 |
| Involving the lamina propria | Destruction of 2/3 of the basal crypts | Moderate | Moderate | Moderate | 26-50% | 2 |
| Involving the submucosa | Destruction of basal crypts with intact epithelium | Severe | Severe | Severe | 51-75% | 3 |
| Involving the submucosa | Complete destruction of basal crypts and epithelium | Severe | Severe | Severe | 76-100% | 4 |
